# The Interplay between miRNA-Related Variants and Age-Related Macular Degeneration: EVIDENCE of Association of *MIR146A* and *MIR27A*

**DOI:** 10.3390/ijms20071578

**Published:** 2019-03-29

**Authors:** Claudia Strafella, Valeria Errichiello, Valerio Caputo, Gianluca Aloe, Federico Ricci, Andrea Cusumano, Giuseppe Novelli, Emiliano Giardina, Raffaella Cascella

**Affiliations:** 1 Molecular Genetics Laboratory UILDM, Santa Lucia Foundation, 00142 Rome, Italy; valeriaerrichiello@hotmail.it (V.E.); v.caputo91@gmail.com (V.C.); emiliano.giardina@uniroma2.it (E.G.); raffaellacascella@virgilio.it (R.C.); 2 Department of Biomedicine and Prevention, Tor Vergata University, 00133 Rome, Italy; novelli@med.uniroma2.it; 3 UOSD Retinal Pathology PTV Foundation Policlinico Tor Vergata University, 00133 Rome, Italy; gianluca.aloe@alice.it (G.A.); rccfrc00@gmail.com (F.R.); cusumano@tin.it (A.C.); 4 Department of Biomedical Sciences, Catholic University Our Lady of Good Counsel, Tirana 1000, Albania

**Keywords:** AMD, miRNAs, epigenetics, susceptibility, inflammation, choroidal neovascularization, neurodegeneration

## Abstract

The complex interplay among genetic, epigenetic, and environmental variables is the basis for the multifactorial origin of age-related macular degeneration (AMD). Previous results highlighted that single nucleotide polymorphisms (SNPs) of *CFH*, *ARMS2*, *IL-8*, *TIMP3*, *SLC16A8*, *RAD51B*, *VEGFA*, and *COL8A1* were significantly associated with the risk of AMD in the Italian population. Given these data, this study aimed to investigate the impact of SNPs in genes coding for MIR146A, MIR31, MIR23A, MIR27A, MIR20A, and MIR150 on their susceptibility to AMD. Nine-hundred and seventy-six patients with exudative AMD and 1000 controls were subjected to an epigenotyping analysis through real-time PCR and direct sequencing. Biostatistical and bioinformatic analysis was performed to evaluate the association with susceptibility to AMD. These analyses reported that the SNPs rs11671784 (*MIR27A*, G/A) and rs2910164 (*MIR146A*, C/G) were significantly associated with AMD risk. Interestingly, the bioinformatic analysis showed that MIR27A and MIR146A take part in the angiogenic and inflammatory pathways underlying AMD etiopathogenesis. Thus, polymorphisms within the pre-miRNA sequences are likely to affect their functional activity, especially the interaction with specific targets. Therefore, our study represents a step forward in the comprehension of the mechanisms leading to AMD onset and progression, which certainly include the involvement of epigenetic modifications.

## 1. Introduction

The network of interactions between genetic and non-genetic factors has been so far investigated in several multifactorial disorders, including neurodegenerative, cardiovascular, and retinal diseases [1]. Interestingly, the prevalence of these pathologies is directly correlated to the progressive ageing of populations, which represents a common triggering factor associated with the onset and evolution of complex disorders [1,2,3]. In this context, the advancement of biomedical research and the application of artificial intelligence systems have been crucial to elucidate the impact of genes, epigenetic modifications, aging, nutrition, drugs, microbiomes, and other environmental factors on health and disease [2]. Such an experimental approach has been very successful in the study of age-related macular degeneration (AMD). In fact, a large number of population-based studies on AMD have identified several genetic and non-genetic variables affecting susceptibility and disease outcome [4,5,6,7]. Furthermore, previous results have highlighted prominent differences concerning genetic and non-genetic contributors to AMD within the Italian cohort compared to worldwide populations [8,9,10,11]. Among genetic variables, single nucleotide polymorphisms (SNPs) of *CFH*, *ARMS2*, *IL-8*, *TIMP3*, *SLC16A8*, *RAD51B*, *VEGFA,* and *COL8A1* are significantly associated with a higher risk of AMD in the Italian cohort, accounting for the 23% of disease susceptibility, in contrast to the 46–71% observed across global populations [8,12]. As expected, non-genetic factors (age, diet, and smoking habit) are associated with AMD, covering the 10% of disease susceptibility in the Italian population, in contrast to the 19–37% reported in other cohorts [8,12]. The analysis of gene–gene and gene–environment interactions revealed that AMD-associated genes may be involved in the induction of angiogenesis; alteration of extra-cellular matrix (ECM) remodeling mechanisms and of Bruch’s Membrane (BrM) integrity and permeability; modification of retinal pigment epithelium (RPE) and photoreceptor cell activities; and over-activation of inflammatory and immune responses [1,5,8,13]. The alteration of these mechanisms helps exacerbate the damage caused by aging and environmental factors, leading thereby to the onset and the advancement of AMD. Altogether, these data provided an overall picture of AMD susceptibility in the Italian population, composed of genetic contributors (23%) and non-genetic contributors (10%). The remaining 67% is still a matter of investigation. In this perspective, epigenetic modifications represent the most promising factors, given their ability to modulate gene expression in response to external stimuli without modifying the DNA sequence. In particular, epigenetics can operate through DNA methylation, histone modification (acetylation and deacetylation), chromatin remodeling, and non-coding RNA-mediated gene silencing (miRNAs), which altogether create and maintain a heritable chromatin structure and allow access to nuclear transcription factors [1]. Among all, MiRNAs are the mostly investigated in complex disorders, given their function throughout the human genome and different tissues. MiRNAs consist of small 22-mer oligonucleotides that regulate gene expression by targeting specific mRNA. More than 60% of human genes contain conserved miRNA binding sites, although structural and sequence variants are known to affect the miRNA–mRNA affinity (by creating, disrupting, or altering the miRNA specific binding ability). In this context, most of the variants are SNPs localized either within the sequence, encoding the miRNAs, or within the 3′-UTR of their target genes. These variants essentially impact the transcriptional profile of target genes as well as the miRNA–mRNA interactions [14]. Given these premises, the present work aimed to study the genes coding for MIR146A, MIR31, MIR23A, MIR27A, MIR20A, and MIR150 in order to search for variants contributing to AMD. These miRNAs were selected on the basis of literature data concerning their role of in AMD etiopathogenesis, especially those involved in angiogenic, inflammatory, and cell survival processes in response to external stimuli (oxidative stress, ageing, and nutrient intake) [13,15].

## 2. Results

The screening analysis on the initial subset of samples highlighted the presence of following polymorphisms: rs2910164 (C/G, *MIR146A*); rs772646842 (G/A, *MIR31*); rs149347978 (C/T, *MIR31*); rs192240130 (T/C, *MIR31*); rs771610178 (G/C, *MIR23A*); rs11671784 (G/A, *MIR27A*); rs895819 (T/C, *MIR27A*); and rs138052193 (-/AG, *MIR150*). The analysis of *MIRNA20A* did not report any variants, resulting in completely wild-type both in case and control subjects. Concerning rs138052193, the association analysis cannot be applicable because the frequency distributions did not observe the Hardy–Weinberg equilibrium. Statistical association was significant (*p* < 0.05) for three SNPs within *MIR146A* (rs2910164, C/G) and *MIR27A* (rs11671784, G/A; rs895819, T/C), respectively (Table 1). The computation of OR revealed that the variant alleles of the SNPs were associated with an increased susceptibility to AMD with respect to wild-type alleles (Table 1).

A statistical association analysis was also performed considering the genotypes of the risk variants. As shown in Table 2, the association was significant for the rs2910164 and rs11671784 genotypes, reporting a higher susceptibility to AMD for heterozygous and homozygous variant classes. Concerning rs11671784, the precise OR value cannot be computed for the AA genotype because was not found in our control subjects. The association analysis was not significant for rs895819. Moreover, the evaluation of dominant and recessive models highlighted a positive association with genotypes carrying at least one risk allele of rs2910164 and rs11671784 (Table 2). Even in this case, the OR for the rs11671784 recessive model cannot be reliable because of the lack of homozygous variant genotypes.

**Figure 1:**
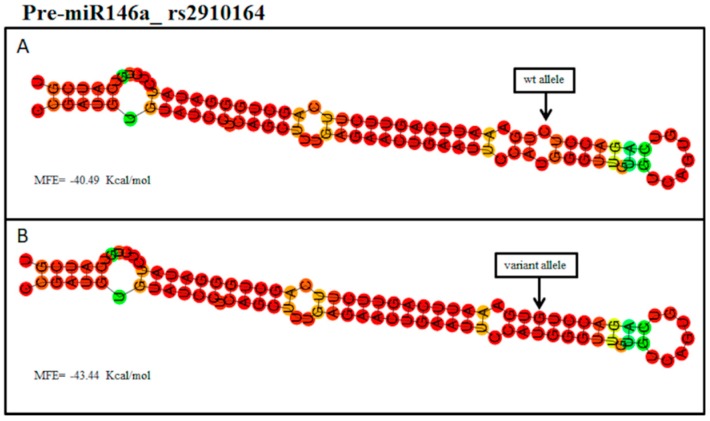
(**A**) Predicted hairpin structure of the pre-miR-146a with the rs2910164_wild type allele (C). (**B**) Predicted hairpin structure of the pre-miR-146a with the rs2910164_variant allele (G). The computed minimum free energy (MFE) of the thermodynamic ensemble is reported. The position of the SNP is shown by the arrow. wt: wild-type.

Bioinformatic analysis reported that MIR146A binds different target genes, including *CFH*, *IL-6*, *HTRA2,* and *IRAK1*. Interestingly, rs2910164 maps within the seed sequence of MIR146A, suggesting that the presence of the variant allele could alter the miRNA–mRNA binding affinity. Moreover, the interrogation of PolymiRTS reported that the variant allele (G) of rs2910164 may disrupt the binding site for *IL-6* and create new ones for *HTRA2* and *IRAK1*. The Vienna RNAFold algorithm allowed us to predict the impact of wild-type and variant sequences of *MIR146A* on the secondary structure of the pre-miRNAs (hairpin structure). In particular, the sequence containing the variant allele (G) of rs2910164 (*MIR146A*) showed a pre-miRNA secondary structure characterized by a lower minimum free energy (MFE = −43.44 Kcal/mol) with respect to the hairpin structure predicted with the wild-type sequence (containing the C allele, MFE = −40.49 Kcal/mol) (Figure 1).

**Figure 2:**
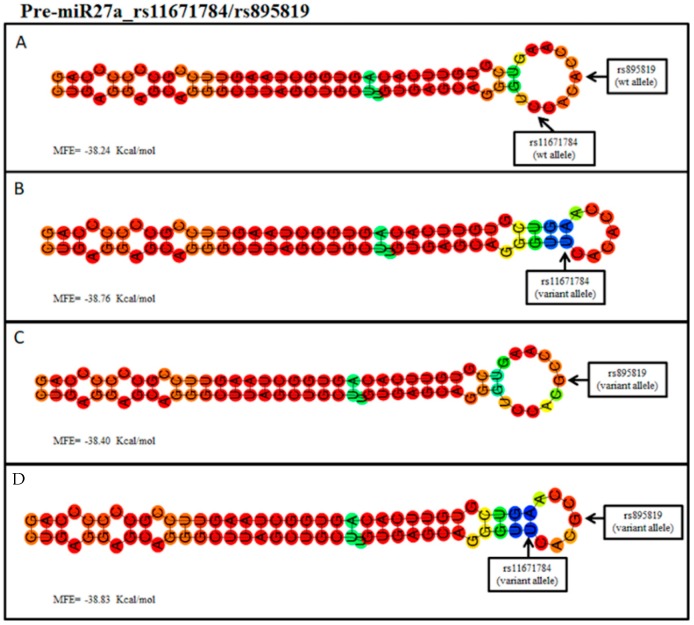
(**A**) Predicted hairpin structure of the pre-miR-27a with the rs11671784 and the rs895819_wild type alleles (C and A, respectively). (**B**) Predicted hairpin structure containing the rs11671784_variant allele (T). (**C**) Predicted hairpin structure containing the rs895819_variant allele (G). (**D**) Predicted hairpin structure with both the rs11671784 and the rs895819_variant alleles (T and G, respectively). The computed minimum free energy (MFE) of the thermodynamic ensemble is reported. The positions of the single nucleotide polymorphisms (SNPs) are shown by the arrow. The alleles are coded considering the MIR27A strand. wt: wild-type.

Successively, the analysis was extended on the variants located within *MIR27A*. Interrogation of bioinformatic tools revealed that miR27A binds different targets, such as *SEMA6A*, *APBB2*, *VEGFC*, *SPROUTY2*, and *PPARγ* genes. Concerning the rs11671784 in *MIR27A*, the hairpin structure derived by the sequence with the variant allele (A) generated a secondary structure with an MFE = −38.76 Kcal/mol, whereas the wild-type structure (with G allele) showed an MFE = −38.24 Kcal/mol (Figure 2A,B). The prediction of secondary structures concerning rs895819 reported that the sequence carrying the variant allele (C) may create a structure with an MFE = −38.40 Kcal/mol (Figure 2C). Given that both SNPs were located in *MIR27A*, the prediction was also performed on the sequence containing both the variant alleles of rs11671784 and rs895819, obtaining an MFE = −38.83 Kcal/mol (Figure 2D).

Altogether, these results suggest that the different MFE of the thermodynamic ensemble detected in the variant structures may enhance the stability of the pre-miRNA-146a and pre-miRNA-27a and the subsequent processing into the mature miRNAs. However, the MFE was found to be much more different in miR146a variant structures compared to miR27a (−2.95 Kcal/mol vs −0.5 Kcal/mol, respectively), suggesting that polymorphisms of MIR146A and MIR27A may affect the processing into mature miRNAs at different levels. Their variable impact is likely to be directly related to the positioning of the SNPs within the pre-miRNA sequence. In fact, rs2910164 is localized in the seed sequence of the pre-miR-146a and, in turn, may influence the miRNA binding affinity with their targets, whereas rs11671784 and rs895819 are situated in the terminal loop of the pre-miR27a, suggesting that they may influence the expression levels of mature miR27a without substantially impair its processing and binding affinity with target mRNAs.

## 3. Discussion

The extensive research concerning the role of miRNAs as regulatory elements affecting gene expression sheds light on the possible contribution of epigenetics to health and disease. In particular, the main function of miRNAs consists in their ability to bind specific target mRNAs, inducing their translational repression or degradation in response to external stimuli. During the study of miRNAs expression profile, increasing evidence proved that polymorphisms within the DNA sequence encoding miRNAs can modify their transcription and binding affinity with the corresponding target mRNAs. In this context, the presence of SNPs regulating miRNA–mRNA interaction suggested the existence of a “genetics of epigenetics” contributing to the onset and progression of complex disorders. Therefore, the present study aimed to investigate the variability of a set of genes coding for candidate miRNAs involved in molecular pathways leading to AMD etiopathogenesis. Given these premises, MIR146A, MIR31, MIR23A, MIR27A, MIR20A, and MIR150 were selected for genotyping analysis on a cohort of 1976 Italian subjects. Three polymorphisms were found to be significantly associated with AMD, namely, rs2910164 (*MIR146A*), rs11671784 (*MIR27A*), and rs895819 (*MIR27A*). These association data, together with bioinformatic results, suggested that both MIR146A and MIRNA27A may be implicated in AMD etiopathogenesis. In particular, up-regulated levels of miRNA146a expression have been found in the plasma and vitreous humor of patients with AMD as well as plasma and cerebrospinal fluid (CSF) of patients affected with Alzheimer’s Disease (AD) [16]. On this subject, *CFH* has already been described in relation to MIR146A as an epigenetic modulator of *CFH* expression in brain and retina. Up-regulated levels of MIR146A have been associated with decreased levels of *CFH*, *IL-6*, *IRAK1*, and *TRAF6* expression, suggesting that it may contribute to the alteration of innate immune response and neuroinflammation in degenerating human brain and ocular tissues [17]. In this context, the presence of rs2910164 in the seed sequence of MIR146A may alter its binding affinity with its mRNA targets, as shown by the disruption of *IL-6* binding site and the creation of new binding sites for *IRAK1* and *HTRA2*. The homonymous proteins encoded by *IL-6* and *IRAK1* have been investigated as two key modulators of inflammation together with IL-8 [18]. Both IL-6 and IRAK1 have been proposed as potential targets ofMIR146A, which thereby may be able to control the activation of NF-kb pathway and the related immune/inflammatory responses occurring in ageing processes and age-related disorders, including AMD [18,19]. Interestingly, *HTRA2* is supposed to be involved in the death of RPE cells occurring in AMD, by inducing apoptosis under cellular oxidative stress and disrupting mitochondrial homeostasis [20]. This gene belongs to the HTRA serine protease family, including *HTRA1*, which is one of the first loci to be associated with AMD because of the strong linkage disequilibrium (LD) with *AMRS2*. In addition, *HTRA2* has been linked to Parkinson’s Disease (PD), suggesting that it may represent a common triggering factor of both disorders [21].

The prediction analysis performed with the RNAfold algorithm revealed a −2.95 Kcal/mol difference in the MFE of the thermodynamic ensemble of the structure predicted for the variant pre-miRNA-146a compared to wild type. This analysis indicated that the variant allele may enhance the stability of the pre-miRNA-146a hairpin and, consequently, its processing into the mature MIR146A. Altogether, these results suggest that the rs2910164 is likely to modify the binding affinity and the final production of mature MIR146A. As a result, variants of *MIR146A* may alter the interaction with mRNA targets (especially, *CFH*, *IL-6*, *IRAK1*, and *HTRA2*) and, consequently, contribute to exacerbate inflammatory signaling and immune response over activation typical of AMD. Interestingly, the uncontrolled regulation of these molecular mechanisms has also been related to the variability of genes (namely, *CFH*, *ARMS2/HTRA1, IL-6*, *IL-8*, *COL8A1*, *SLC16A8*, and *VEGFA*) associated with a specific susceptibility to exudative AMD [1].

Given its modulatory effect on immune and inflammatory response, *MIR146A* polymorphisms have also been associated with several other disorders, including cancer, psoriasis, psoriatic arthritis, diabetes, and cardiovascular disorders [22,23,24,25,26]. The dynamic interactive roles of MIR146A could also be useful to investigate the relationship between AMD and the co-occurrence of late-onset disease conditions, especially cardiovascular diseases, autoimmune diseases, diabetes, chronic kidney disease, AD, and PD [1,27].

Over *MIR146A*, SNPs of *MIR27A* (rs11671784 and rs895819) were significantly associated with AMD in our cohort. *MIR27A* encodes the homonymous miRNA, which is expressed in highly vascularized tissues and is involved in cell cycle regulation, proliferation, apoptosis, and differentiation [28]. MIR27A has been found to be up-regulated in choroidal neovascularization (CNV), acute lymphoblastic leukemia, acute myeloid leukemia, and hepatocarcinoma [28,29]. This relationship may be explained by the fact that MIR27A seems to play a key role in angiogenesis. In fact, it may promote the proliferation and migration of endothelial cells by directly binding and repressing SPROUTY2 and SEMA6A target proteins, which normally act as anti-angiogenic factors by exerting a negative regulation of the VEGF-mediated signaling [29]. On this subject, the prediction analysis performed with the RNAfold algorithm revealed a very small difference (~0.5 Kcal/mol) in the MFE of the thermodynamic ensemble of the pre-miRNA-27a structure predicted for both the associated variants (rs11671784 and rs895819) with respect to the wild type. This analysis indicated that these variant alleles have a very slight impact on the stability of the pre-miRNA-27a hairpin and, consequently, its processing into the mature MIR27A. However, these polymorphisms may still modify the expression levels of mature miR27a, resulting in a different modulatory effect on its mRNA targets, as shown by the studies performed on gastric cancer susceptibility [30,31]. Bioinformatic analysis revealed supporting data concerning the possible association between MIR27A and AMD. In fact, MIR27A was shown to bind several target genes, including *SEMA6A*, *VEGFC*, *APBB2*, and *PPARγ*. These results suggest that the interaction with *SEMA6A* and *VEGFC* may contribute to the activation of angiogenic pathways specific of exudative AMD. In this context, it is important to remark that *VEGFA*, *IL-8*, and *COL8A1* genes have also been indicated as key regulators of angiogenic mechanisms and have been associated with a higher susceptibility to AMD [1,5,13]. On the other hand, *APBB2* has been extensively described in relation to its association with late-onset AD because of its role in processing amyloid precursor protein (APP) into β-amyloid [32]. Recent evidence hypothesized that APBB2 production may increase the formation and deposition of toxic β-amyloid both in neuronal and retinal cells [33,34]. In this perspective, the potential interaction between MIR27A and *APBB2*-related mRNA may contribute to the activation of etiopathogenetic pathways shared in AMD and neurodegenerative disorders. The possible relationship between AMD and the MIR27A-mediated regulation of *PPARγ* expression may be explained by the anti-inflammatory/anti-angiogenic role of *PPARγ* as inhibitor of VEGF expression in exudative AMD [35]. *PPARγ* dysregulation has been reported in several disorders characterized by over-activation of inflammatory response, including obesity, diabetes, atherosclerosis, and cancer [28].

**Figure 3:**
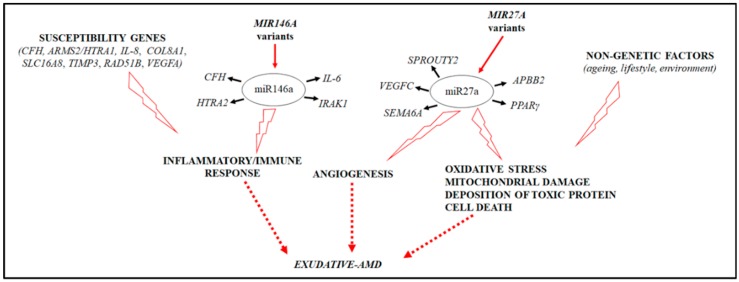
The figure illustrates the interplay between *MIR146A*, *MIR27A* variants, and AMD etiopathogenetic pathways.

Altogether, these data suggested that polymorphisms in *MIR27A* and *MIR146A* may finally contribute to the exacerbation of angiogenic and inflammatory pathways underlying AMD etiopathogenesis (Figure 3). However, functional assays are necessary to validate the real impact of rs2910164, rs11671784, and rs895819 on the biogenesis, transcription, and function of mature MIR146A and MIR27A.

In conclusion, the present study represents a step forward in the comprehension of the mechanisms leading to AMD onset and progression, which certainly include the involvement of epigenetic modifications. The availability of epigenetic biomarkers such as miRNAs could be crucial to better understand the main signatures influencing the individual risk profile for exudative AMD. In this context, epigenetic profiles should be combined with AMD-specific genetic and non-genetic features in order to create a web-based platform addressed to provide patients with preventative and precision medicine strategies [36,37].

## 4. Material and Methods

### 4.1. Study Cohort and DNA Extraction

This study enrolled 1976 individuals, including 976 exudative-AMD cases and 1000 control subjects. Cases were selected from the Ophthalmology Unit of the PTV General Hospital of Rome, U.O. Oculist Foundation IRCCS “Cà Granda” Maggiore General Hospital of Milan, Department of Clinical Physiopathology of the University of Turin, and Department of Clinical Science of Sacco Hospital of Milan. The control subjects were recruited from the UOSD SIMT and the Ophthalmology Unit of the PTV General Hospital of Rome. Clinical data referred to the recruited subjects are summarized in Table 3. The study was approved by the Ethics Committee of the “Tor Vergata”, University of Rome (reference number: 16.15, approved on 23 January 2015). The study was performed according to the Declaration of Helsinki, and all participants provided signed informed consent. Blood samples were obtained from all subjects in order to extract genomic DNA. Genomic DNA extraction was performed using the EZ1 Advanced XL automated extractor and the EZ1 DNA Blood 200 µL Kit (Qiagen, Valencia, CA, USA) according to manufacturer’s instructions.

### 4.2. Genotyping Analysis

Initially, a subset of samples was subjected to a screening analysis for the research of candidate variants (SNPs) located within *MIR146A* (5q33.3); *MIR31* (9p21.3); *MIR23A* (19p13.12); *MIR27A* (19p13.12); *MIR20A* (13q31.3); and *MIR-150* (19q13.33). The selection of the miRNAs to be studied was performed on the basis of literature data and their target mRNAs predicted by bioinformatic analysis. Prominent attention was given to miRNAs targeting genes that are known to be associated with exudative-AMD or biological pathways involved in disease onset and progression. The screening was performed by direct sequencing with BigDye Terminator v3.1 and BigDyeXTerminator kit according to the manufacturer’s instructions. Samples were run by capillary electrophoresis on ABI3130xl (Applied Biosystems, Warrington, UK) and analyzed by sequencing analysis (version 5.3, Applied Biosystems, Foster City, CA, USA). Successively, the identified variants were genotyped on the whole study cohort, utilizing TaqMan chemistry and a 7500 fast real-time PCR device according to the manufacturer’s instructions (Applied Biosystems, Warrington, UK). The genotyping results were interpreted using Sequence Detection System 2.1 software (Applied Biosystems, Warrington, UK). Each real-time PCR run was performed using a negative control and three positive control samples previously confirmed by direct sequencing.

### 4.3. Biostatistical and Bioinformatic Analysis

The genotyping results were subjected to biostatistical analysis to evaluate associations with AMD. First, the genotyping data reported in our cohort were tested to confirm the Hardy–Weinberg equilibrium (*p* > 0.05). Afterwards, the association between the genotyped SNPs and AMD was measured by calculating the *p*-value (*p*). The statistical associations were considered significant when *p* < 0.05 based on the 95% confidence interval. The strength of the associations was determined by calculating the odd ratio (OR). All the statistical analyses were performed using the SPSS program, version 23 (IBM Corp, Armonk, NY, USA).

Bioinformatic analysis was performed to select and annotate the miRNAs of interest and evaluate the secondary structure of the corresponding pre-miRNAs, the MFE, and the molecular pathways in which the associated miRNA may be involved. To this purpose, miRbase, microRNA.org, and TargetScanHuman tools were used to select and annotate the miRNAs of interest and their related mRNA targets [38,39,40]. PolymiRTS tool (version 3.0, University of Tennessee Health Science Center, Memphis, USA) is a database that gathers polymorphisms (SNPs and indels) in microRNA (miRNA) seed regions and miRNA target sites that may affect miRNA–mRNA interactions and, in turn, miRNA-mediated gene expression [41]. In this work, PolymiRTS database was utilized to evaluate the in silico impact of miRNA variants detected by screening analysis. The Vienna RNAfold algorithm (ViennaRNA package 2.0) was utilized to predict the secondary structures of pre-miRNAs (hairpin structure) and compute the MFE of the thermodynamic ensemble (∆G) [42]. In particular, wild-type and variant miRNA sequences were tested by RNAfold tool in order to evaluate differences in MFE that could affect miRNAs biogenesis and potential binding affinity. Normally, the pre-miRNA structure with lower MFE is expected to be thermodynamically more stable and enhance the processing of the mature miRNA.

## Figures and Tables

**Table 1:** Biostatistical results concerning the association of the variants identified by sequencing of the genes coding for the miRNAs of interest. ns: not significant; na: not available. Significant results are highlighted in bold.

Analyzed Variants	Genotypes Number and Frequencies (%) in Cases	Genotypes Number and Frequencies (%) in Controls	Allelic *p*-Value	Allelic OR (CI:95%)	Impact
***MIR146A*** **rs2910164 (C/G)**	C/C: 57 (7%)	C/C: 100 (10%)	**0.005**		**Risk**
	G/C: 303 (37%)	G/C: 388 (39%)		**G = 1.23 (1.06–1.43)**	
	G/G: 456 (56%)	G/G: 500 (50%)			
*MIR31* rs772646842 (G/A)	G/G: 921 (99%)	G/G:977 (99%)	ns		-
	G/A: 10 (1%)	G/A: 10 (1%)			
	A/A: 0 (0%)	A/A:0 (0%)			
*MIR31* rs149347978 (C/T)	C/C: 921 (99%)	C/C:977 (99%)	ns		-
	C/T: 10 (1%)	C/T: 10 (1%)			
	T/T: 0 (0%)	T/T:0 (0%)			
*MIR31* rs192240130 (T/C)	T/T: 873 (99%)	T/T:922 (98%)	ns		-
	T/C: 9 (1%)	T/C: 18 (2%)			
	C/C: 0 (0%)	C/C: 0 (0%)			
*MIR23A* rs771610178 (G/C)	G/G: 975 (99%)	G/G: 940 (99%)	ns		-
	G/C: 10 (1%)	G/C: 10 (1%)			
	C/C: 0 (0%)	C/C: 0 (0%)			
***MIR27A*** **rs11671784 (G/A)**	G/G: 742 (93%)	G/G: 768 (97%)	**0.001**		**Risk**
	G/A: 50 (6%)	G/A: 24 (3%)		**A = 2.29 (1.41–3.72)**	
	A/A: 2 (1%)	A/A: 0 (0%)			
***MIR27A*** **rs895819 (T/C)**	T/T: 399 (52%)	T/T: 495 (57%)	**0.03**		**Risk**
	T/C: 301 (39%)	T/C: 315 (36%)		**C = 1.19 (1.02–1.39)**	
	C/C: 67 (9%)	C/C: 60 (7%)			
*MIR20A* (completely wild-type)	-	-	na		-
*MIR150* rs138052193 (-/AG)	541 (62%)	608 (67%)	na		-
	323 (37%)	272 (30%)			
	9 (1%)	27 (3%)			

**Table 2:** Statistical association analysis considering the genotypes of the risk variants and dominant and recessive models. ns: not significant, na: not available. Significant results are highlighted in bold.

Analyzed Variants	Genotype *p*-Value	Genotypic OR (CI:95%)	Dominant Model OR (CI:95%)	Recessive Model OR (CI:95%)
***MIR146A*** **rs2910164 (C/G)**	**0.019**	**GC = 1.38 (1.00–1.96)** **GG = 1.60 (1.12–2.26)**	**1.50 (1.10–2.10)**	**1.23 (1.02–1.48)**
***MIR27A*** **rs11671784 (G/A)**	**0.003**	**GA = 2.15 (1.31–3.54)** **AA =** na	**2.24 (1.36–3.67)**	na
*MIR27A* rs895819 (T/C)	ns	-	-	-

**Table 3:** Collection of data concerning the subjects enrolled in the study.

Data	Cases	Controls
Age	±77 years old	±72 years old
Sex	F: 54% M: 46%	F: 56% M: 44%
Type of CNV	Type 1:53% Type 2: 47%	-
